# Wall Mosaics: A Review of On-Site Non-Invasive Methods, Application Challenges and New Frontiers for Their Study and Preservation

**DOI:** 10.3390/jimaging6100108

**Published:** 2020-10-12

**Authors:** Antonina Chaban, Rita Deiana, Vivi Tornari

**Affiliations:** 1 National Institute of Optics, Italian National Research Council, 50125 Florence, Italy; 2 Department of Cultural Heritage, University of Padova, Piazza Capitaniato 7, 35139 Padova, Italy; rita.deiana@unipd.it; 3 Institute of Electronic structure and Laser, Foundation for Research and Technology Hellas, N. Plastira 100, 70013 Voutes, Crete, Greece; vivitor@iesl.forth.gr

**Keywords:** non-invasive, non-contact, wall mosaics, cultural heritage, diagnostics

## Abstract

This review concerns the challenges and perspectives of on-site non-invasive measurements applied to wall mosaics. Wall mosaics, during the centuries, decorated numerous buildings, nowadays being part of world cultural heritage. The preservation and maintenance of these valuable decorations are undoubtedly directly dependent on identifying possible problems that could affect their hidden structure. On-site non-invasive methods, using different contact or no-contact technologies, can offer support in this specific field of application. The choice of the appropriate technique or combination of different techniques depends, in general, on the depth of investigation, the resolution, the possibility to have direct contact with the surfaces or, on the contrary, limited accessibility of the wall mosaics due to their location (e.g., vaults), as well as deterioration problems, (e.g., voids, detachments, or humidity effects). This review paper provides a brief overview of selected recent studies regarding non-invasive methods applied to the analysis of wall mosaics. This review, discussing the assessment of advantages and limitations for each method here considered, also considers possible future developments of imaging techniques in this specific context for cultural heritage applications.

## 1. Introduction

The use of wall mosaics as surface decoration in historical buildings (e.g., houses, palaces, churches etc.) can be traced back to the ancient Orient using wall coverings made by clay cones, different both in size and thickness from the traditional wall mosaics [1]. The extensive use of the most known small tesserae, as a decoration of walls, columns, pillars, ceilings, and vaults, is documented over the centuries, particularly in Greek, Roman, and Byzantine periods [1,2,3,4,5,6,7,8,9,10], in numerous examples of buildings, which are part of the world cultural heritage. In general, the mosaics located on walls, domes, and vaults are more difficult to study (due to their position, geometric and surface features etc.).

The possibility to quickly and non-invasively identify any anomaly that may compromise the integrity of these decorations, as well as the ability to recognize previous restorations, replacements, and other changes that have occurred over time, in a preventive, non-invasive manner, is crucial in the context of the interventions for their conservation and protection. This review is focused on the main methods currently used and now available for the non-invasive analysis and monitoring of ancient wall mosaics. In general, these decorations below the tesserae present simple preparation layers, compared to the floor mosaics. Moving from the surface towards the wall support, we have the tesserae, generally made of glass, stone, nacre, or ceramic, then the first layer of lime mortar and aggregates of different types (sand, pozzolana, marble dust, fragments of straw etc.) and another layer of bedding mortar, with or without aggregates [3,4]. Differences in the composition, number, and thickness of layers allow for identifying contributions by different mosaic workers, or, more generally, the period of its realization. Among the various problems that may affect the conservation of this type of decoration, there are the alterations of the surface of the tesserae, the presence of voids and inhomogeneities in the underlying layers of mortar. These can lead to the detachment and consequent fall and loss of tesserae. Other problems are related to humidity (e.g., moisture infiltration) that can also generate detachments and efflorescences. In the last decade, many advances have been made in the applications of on-site non-invasive measurements to the analysis of cultural heritage, supporting restoration works and studies aimed at understanding the transformations that may have affected the historical structures over the centuries [11,12,13,14,15,16]. In order to detect or to predict damage, efficient diagnosis and monitoring tools are essential. In particular, the possibility offered by on-site non-invasive measurements to preliminarily analyze the decorations as wall mosaics, identifying any problems and driving the conservation and restoration interventions, opens up new horizons in the field of heritage protection. This ability offered today by some specific methods in the identification of detachments (e.g., using IR thermography) or areas altered/compromised by previous restoration interventions (e.g., presence of different mortars or concrete, metal reinforcements, e.g., using ground-penetrating radar), and the detection of the presence and extent of any water leaks and infiltration (e.g., using electrical resistivity tomography, ground-penetrating radar, IR thermography), therefore, represents the critical issue in the new concept of cultural heritage preservation.

## 2. Wall Mosaic Characteristics and Main Diagnostic Challenges

Although each study case is different, the three fundamental challenges in wall mosaic diagnostics are represented by their complex, layered structure, presence of inhomogeneities (e.g., nails), and location (limited accessibility).

### 2.1. Structural Layering

**Figure 1:**
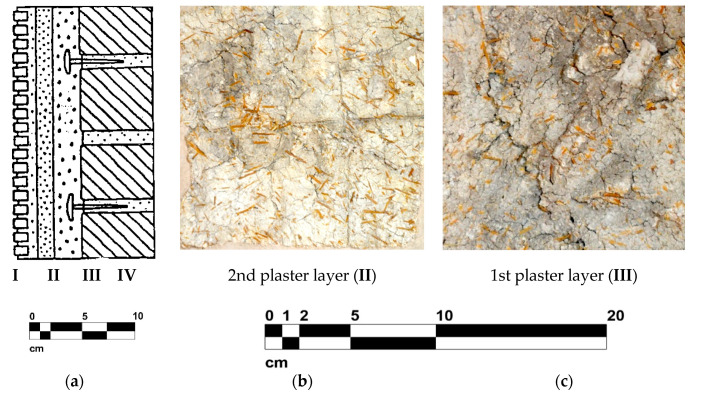
Structural layering of Byzantine wall mosaics: (**a**) section, IV—wall with protruding nails, III—1st plaster layer; II—2nd plaster layer; I—3rd plaster layer and tesserae, after [3]; (**b**) 2nd substrate layer, consisting of lime plaster, sand and straw and (**c**) 1st substrate layer, consisting of pure lime and straw (on display at the museum of Hosios Lukas, Greece).

The ancient wall mosaics, in general, are layered structures (Figure 1a), composed in most cases by three main levels: (1) a superficial decoration layer constituted by tesserae; (2) two or more different mortar layers; (3) wall structure made by bricks o stones [1,2,3,4,5,6,7,8,9]. While stone tesserae were mostly used in floor mosaics, glass tesserae dominate in wall mosaics. The most well known and preserved wall mosaics are of the ancient Roman, Greek, and Byzantine medieval periods.

The Byzantine wall mosaics were mostly realized in several stages directly on the wall, covering it by three different thickness layers of lime mortar. The first two layers were commonly made of lime, bricks, and cut straw, while the third contained only lime and marble powder [9].

### 2.2. Presence of Inhomogeneities

Cut straw was sometimes added to improve the binding characteristics and to control the migration of moisture between pores [3,4,9]. To enhance the cohesion between the layers, the first and the second mortar layers presented a rough surface, with deep bumps or ripples made by the tip of the trowel [3,4,9], as shown in Figure 1b,c, creating an irregular subsurface. To ensure the mortar layer (e.g., on vaults), strong steel flat-headed nails were often placed protruding the wall, as shown in Figure 1a. The final (bedding) layer of mortar could contain additives as linseed oil and adhesive substances to ensure the slowing down of mortar setting while often not regularly placing the tesserae. The tesserae were predominantly made of glass-paste, containing metal oxides, or a gold leaf, extensively used for gilded backgrounds, halos, and details of religious scenes in the churches [1,2,3,4,5,6,7,8,9]. Ancient wall mosaics have been often restored and/or reshuffled over the centuries without documentation of these interventions.

### 2.3. Limited Accessibility

Highly valuable historical and archaeological sites are characterized by limited accessibility due to opening hours to visitors or site logistical conditions. Besides, wall mosaics are often located in high, hardly accessible, or inaccessible parts of buildings (domes, vaults etc.). The limitations are represented by the need to use scaffoldings (if available), by the form of the structural support, and by the impossibility to respect the necessary geometry of the acquisitions (distance, access on both sides of the wall etc.).

## 3. Some Examples of Applications of Non-Invasive Methods to Study Wall Mosaics

From a theoretical point of view, there is a wide range of non-invasive methods and related instruments applied in engineering and cultural heritage studies.

This review considers only non-invasive methods useful to the study of wall mosaics and focuses on the developments and experimental setup proposed by the international scientific community interested in this topic. The most documented literature for in-situ non-invasive methods to study wall mosaics regards ground penetrating radar (GPR) and infrared thermography (IRT) and sporadic example of the use of electrical resistivity tomography (ERT), acoustic methods (e.g., sonic and ultrasonic tests), microwave reflectometry (MWR), and fiber optics microscopy (FOM). More recent literature also reports promising results of laboratory tests by different methods, e.g., electro-optic holography, also called electronic speckle pattern interferometry (ESPI), as well as recent interesting complementary approaches, using digital holographic speckle pattern interferometry and stimulated infrared thermography (DHSPI-SIRT), holographic subsurface radar (HSR), and scanning laser doppler vibrometry (SLDV). The main advantages, limitations, and potentials of each method are discussed below.

### 3.1. Ground Penetrating Radar (GPR)

The use of high-frequency GPR antennas (2 GHz) increased in the last two decades in the field of cultural heritage inspections [11,12,13,14,15,16,17,18,19,20,21,22], with some examples also including wall mosaics structure analysis. Based on the propagation of electromagnetic waves, geophysical methods, applied mainly in environmental and archaeological studies, gained popularity also in the cultural heritage field of investigation thanks to its capability for the fast and easy identification of voids and moisture content variations, both related to the contrast of dielectric permittivity. These aspects are crucial in the localization of in-homogeneities, presence of voids, and moisture content mapping and time-lapse monitoring of these phenomena in mosaic wall analysis. On the other hand, despite the fast and easy acquisition of the data, the principal limitation in using this powerful non-invasive method of investigation is represented by the needed contact between the antenna and the investigated surface that often preclude its application. For a more comprehensive analysis of physical principles, data acquisition, and processing of the GPR method, we suggest consulting the specific literature [23,24].

### 3.2. Infrared Thermography (IRT)

The infrared thermography is an on-site no-contact method widely applied to cultural heritage studies [25,26,27,28,29,30,31,32], with documented applications on wall mosaics analysis. The principle of infrared thermography consists of measuring, without contact, all thermal variations detectable on the surface by the infrared radiation. In general, the thermal variations could indicate different materials, including inhomogeneities, under the decoration layer. Although this measurement refers only to the first few centimeters below the decoration, it represents a perfect combination with the GPR method to identify shallow voids (detachments) or humidity, mainly where the contact with the surface is not admitted. Active infrared thermography, or also called stimulated infrared thermography, foresees to induce a minimal thermal excitation artificially to the surface of the investigated mosaic decoration, in order to increase characteristic thermal differences [25]. The use of passive thermography is preferred when the method is applied to cultural heritage objects; nevertheless, it does not always provide efficient results. The accuracy of temperature measurements is limited when the inspected surface consists of various types of materials with different emissivity values and if there are no naturally occurring thermal contrasts, in particular when examining vault/dome mosaics at a long distance.

Natural thermal contrasts are stronger on externally exposed walls and weaker or not detectable on the internal walls of a building. The advantage of active (or stimulated) infrared thermography is its potential to increase the informativity of a thermographic inspection in the absence of natural thermal contrasts, especially for the study of shallow depth detachments and voids. However, the informativity can still be limited due to insufficient maximum thermal excitation (usually, not higher than ΔΤ = 3.5 °C) in low thermal conductivity material in the superficial layer of the wall.

The most significant advantage of infrared thermography is its non-contact, remote control, and full-field investigation technique. The instruments are generally easily portable. The operator can visualize the results, i.e., thermal images, in real-time. Further thermal data processing and analysis for real case studies of wall mosaics are often necessary. For more details about the theory and application of this method, we suggest to see the specific literature [33,34,35].

### 3.3. Complementary On-Site Non-Invasive Methods

The two above-mentioned established techniques can be complemented by other structural diagnostic methods, which are not systematically used in real case studies yet. They can be divided into two groups: (1) on-site non-invasive techniques with documented applications on real wall mosaics, which include seismic methods (sonic and ultrasonic testing) and electrical resistivity tomography (ERT), and (2) non-invasive methods, tested in laboratory conditions but to the authors’ awareness never applied on real case studies yet, including electro-optic holography (ESPI), digital holographic speckle pattern interferometry (DHSPI) applied in combination with stimulated infrared thermography (DHSPI-SIRT), holographic subsurface radar (HSR), scanning laser doppler vibrometry (SLDV).

The principle of acoustic methods lies in the measurement of sonic/ultrasonic wave velocity propagation through the material. The interpretation of the results is based on the analysis of wave propagation velocity, which differs for different kinds of medium and is altered by the presence of cracks, voids, moisture areas etc. [36]. It is suitable for the evaluation of subsurface and deep wall structure. However, it cannot be regularly applied to highly precious surfaces. The main limitations of seismic methods include their manual contact operation and possible invasivity for deteriorated areas, and insufficient resolution for shallow depth levels.

The principle of electrical resistivity tomography lies in the characterization of the subsurface materials in terms of their electrical properties [37,38]. It can create a 3D representation of electrical resistivity distribution inside the examined objects, e.g., walls underneath a mosaic decoration. It provides the possibility to localize humidity (and then the provenience of the water infiltration and rise) or the presence of cracks and voids, as well as with the GPR method. The principal limitation on ERT applicability on wall decorations is its invasivity. Although the use of medical electrodes is possible, commonly to guarantee the better signal to noise ratio, the use of micro-electrodes (nails) is preferred. This invasivity then effectively limits the use of the ERT method in the mosaic wall analysis. Some studies were carried out in order to overcome this limitation, in particular, by limiting the number of mini electrodes. This method, in its current operation mode, still cannot be regularly applied to real wall mosaic decorations.

Innovative optical techniques are of particular interest in the structural evaluation of cultural heritage objects. Interferometric methods were tested during the last decades in laboratory conditions for non-contact full-field wall mosaic subsurface structural evaluation. The first documented application on a mosaic wall model refers to the method called electro-optic holography, or electronic speckle pattern interferometry (ESPI). It is the optical technique that enables interferometric measurements of surface displacements upon induced stress [39,40] and can be considered as a video equivalent of holographic interferometry [41,42].

A highly promising method is digital holographic speckle pattern interferometry (DHSPI). It is a non-invasive full-field, remote control, and non-contact optical technique based on the combination of holographic interferometry (HI) and electronic speckle pattern interferometry (ESPI). The holographic principle of DHSPI consists of recording phase variations of two mutually coherent laser beams at the single wavelength (532 nm), which give rise to interference fringes recording out-of-plane micro-displacement of the surface, with the resolution of 266 nm. The data acquisition is carried out through interferometric comparison of the two states of the surfaces under examination: the reference state and the excited state. For a more comprehensive study of the DHSPI operating principle and various applications, dedicated literature sources are available [41,42,43,44,45,46,47,48]. Digital holographic speckle pattern interferometry (DHSPI) coupled with stimulated infrared thermography (SIRT) proved to be a powerful non-invasive, non-contact, full-field, and remote-control subsurface diagnostics system, already applied to a variety of movable and immovable works of art [42,43,44,45,46,47,48,49,50]. When DHSPI is applied in combination with SIRT, thermal excitation is used for simultaneous acquisition by the two techniques. The differences in surface micro-deformations are induced by differences in the thermal expansion behavior of the materials and elements in the object’s subsurface structure. This technique can be applied to temporal monitoring of structural changes, based on environmental and climatic changes, conservation treatments, natural or stimulated aging, etc. To the authors’ awareness, it has not been applied yet to wall mosaics in-situ. The significant advantages of DHSPI and DHSPI-SIRT include its applicability and informativity, independently from the form and regularity of the surface (it can be applied to non-planar surfaces), even at long distances (up to 15 m). The system provides real-time images visualization, and raw results were already easy to analyze and interpret.

The method called scanning laser doppler vibrometry (SLDV) is used to perform non-contact vibration measurements of a surface [51,52,53]. The surface of an object under investigation is slightly vibrated by mechanical and acoustical actuators, while a laser doppler vibrometer scans the surface velocity and produces velocity amplitude and phase 2D or 3D maps. In the presence of a defect, i.e., a detachment or void, the velocity in the affected area is higher than in the neighboring areas. The Scanning LDV measures surface velocities point by point using interferometric techniques and a couple of galvanometric driven mirrors steering the laser beam. The main advantage of the system for the structural evaluation of wall mosaics lies in its remote control and non-contact operation, high sensibility, and resolution.

The main limitation of ESPI, DHSPI, and SLDV consists of their high sensitivity to surrounding vibrations (movement of operators and other people, other technical and mechanical vibrations).

Holographic subsurface radar (HSR) is an innovative type of a surface penetrating radar, which proved some successful results in structural evaluation on planar surfaces in engineering and built heritage field [54,55,56,57,58]. It operates with continuous-wave, at several close frequencies around 2 GHz, 4 GHz, or 7 GHz, depending on the model. Both the reflected signal and reference signal have the same frequency, but the reflected signal has a phase shift depending on the distance (depth) to the reflector. The holographic principle refers to image formation, and the method can be referred to as microwave holography [50]. The detectability of the target (a defect or an inhomogeneity) within the subsurface is based on contrasts in dielectric permittivity. This technique proved its efficiency in the immediate detection of dielectric contrasts in the subsurface structural evaluation of built heritage, including metallic and plastic elements, moisture infiltrations. The method allows for the estimation of the location depth of the target, as well as the size and geometry of revealed features if dielectric permittivity values are known. The HSR instrument, called Rascan, is lightweight and easily portable. However, the acquisition procedure is manual, requires advanced accuracy and the obtained results depend strongly on the precision of acquisition lines. They can be performed with a minimum possible offset (0.5 cm and an intermediate layer (PVC film or a transparent sheet) is suggested to protect the mosaic surfaces. Repeated acquisitions in two perpendicular directions are recommended. The main applicability limitation is that it encounters difficulties when evaluating irregular or non-planar (for example, curved) surfaces, and currently, the developers of the technique are working on the further developments of the scanning system [59]. For a more comprehensive treatment of the subject, the reader can refer to reliable bibliographic sources [54,55,56,57,58,59].

The results obtained on real case studies and laboratory simulated models by established and innovative methods, presented in scientific literature, are presented below.

### 3.4. In-Situ Results

**Figure 2:**
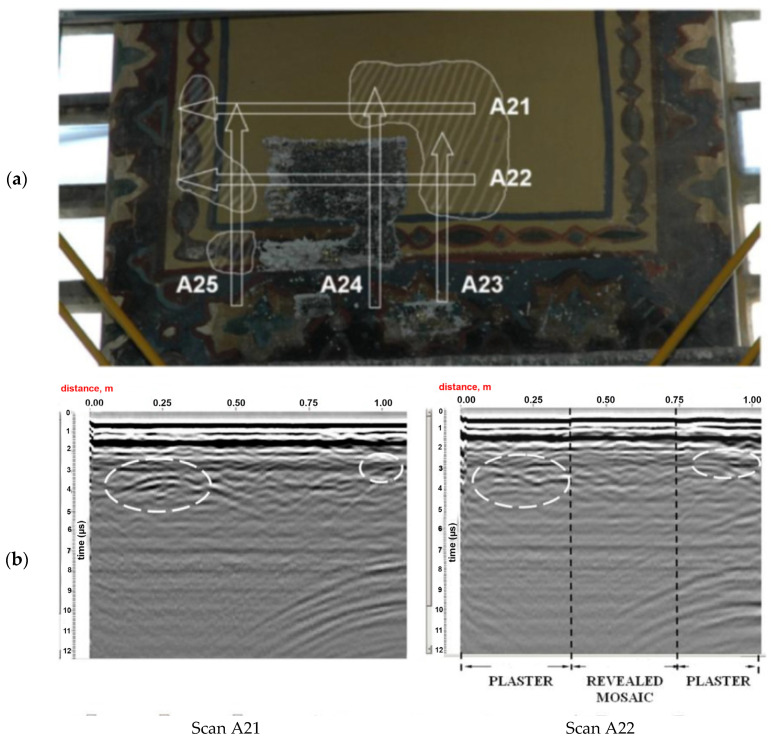
Ground-penetrating radar (GPR) results on wall mosaics in Hagia Sophia (Istanbul, Turkey): (**a**) examined area of a plastered surface; (**b**) GPR scans, the highlighted ripples reveal the presence of plastered mosaics. After Moropolou et al. [62] (reprinted with copyright permission).

**Figure 3:**
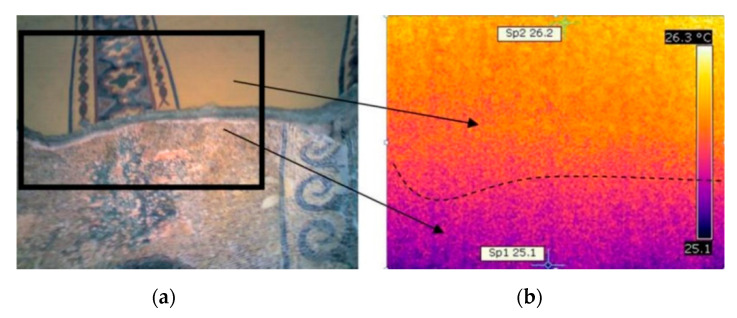
Infrared thermography (IRT) results at the plaster (top)–mosaic (underneath) interface in Hagia Sophia (Istanbul, Turkey): (**a**) visible image and the examined area; (**b**) thermogram showing the temperature difference of a mean value of 1.1 °C between the plastered surface and the mosaic. After Moropolou et al. [62] (reprinted with copyright permission).

Ground-penetrating radar (GPR), infrared thermography (IRT), and fiber optics microscopy (FOM) were applied in combination with wall mosaics in Hagia Sophia (Istanbul, Turkey). The church of Hagia Sophia is decorated with wall mosaics dating back to the fourth century [60]. The church suffered many interventions, damages, earthquakes. After its first conversion to a mosque upon the fall of Constantinople (1453), the original mosaic decoration was plastered over. In 1935, it became a museum, with another conversion into a mosque in 2020. The research issues of the interior mosaics in Hagia Sophia, approached by different expert groups in the last years, vary from revealing hidden layers of covered (plastered) mosaics to the evaluation of complex surface and subsurface structural conditions. The GPR was efficient in revealing the presence of covered mosaic layers (Figure 2). The IRT was not capable of detecting covered mosaic layers; however, it provided complementary information on differences in emissivity properties of different materials (Figure 3). The fiber optics microscope (FOM) was used to assess the conservation state of the decoration layer and bedding mortar, providing detailed complementary information to the assessment of aging and decay of materials due to porosity, cracking, to correlate the revealed water intrusions and the identification of the possible level of moisture in the subsurface beneath the mosaics [61,62,63,64]. The studies conducted since 2000 also included evaluations of compatibility of consolidation interventions [65] and led to a series of advancing experimental laboratory studies [66,67].

Another interesting case study is based on the use of Ground Penetrating Radar (GPR) in combination with seismic techniques. They were applied in-situ on wall mosaics at the Katholikon of Dafni Monastery (Athens, Greece) in 2003. It is an XI century World Heritage monument, famous for its mosaics [68]. After an earthquake in 1999, the monument was heavily damaged, and the subsurface structural investigation is part of a big conservation-restoration campaign. The research issues were the following: to study the internal masonry structure and of the mosaics substrates, to assess the performance of conservation-restoration treatments. In some areas, these techniques were applied before and after hydraulic grouts injections for filling cracks and voids in the shallow depth subsurface and structural support of wall mosaics. High-frequency ground-penetrating radar was successfully applied to the mapping of more than 50 mosaics in order to identify critical areas with delaminations, changes of substrata mortar (i.e., recent repairs), or buried heterogeneities. With the use of seismic tomography technique, as well as by manual sonic investigation by conservators, maps revealing the internal mechanical properties of the masonry walls were produced.

**Figure 4:**
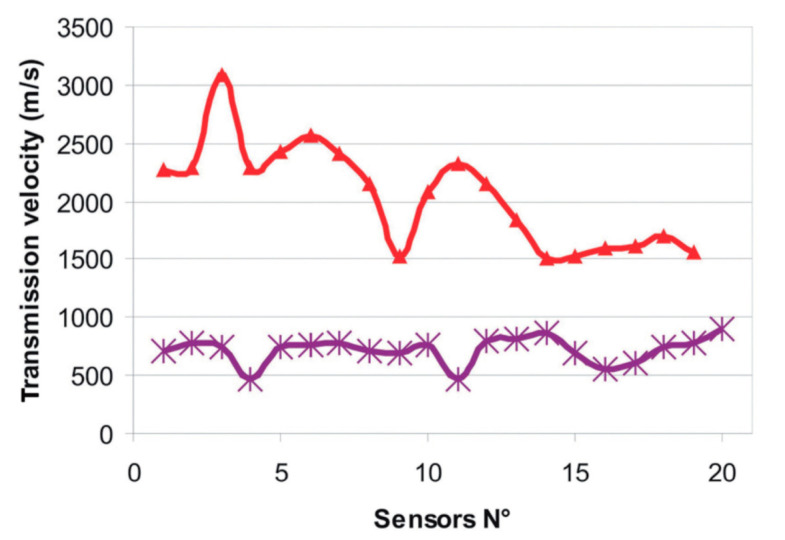
Results of acoustic techniques applied in-situ to the assessment of efficiency of grouting, Katholikon of Dafni Monastery, Athens, Greece. Sonic transmission velocities across a similar wall grouted (red, higher velocity in repaired areas) or not yet grouted (purple, lower velocity in the presence of cracks and voids), showing the assessment capabilities of the technique. After Côte & X. Dérobert et al. [69] (reprinted with copyright permission).

Complementary use of non-invasive techniques showed usefulness in the detection of subsurface structural problems and the efficiency assessment of a grouting intervention [69]. The results based on the assessment sonic transmission velocities of grouted and not grouted areas of a wall are shown in Figure 4.

**Figure 5:**
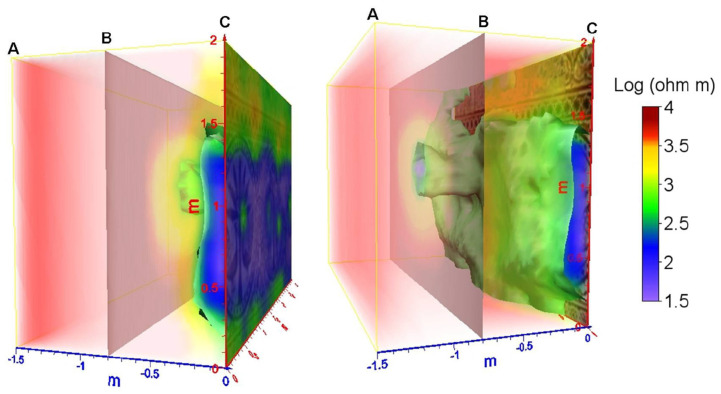
Electrical resistivity tomography (ERT) results in precious wall mosaics, fountain room of the Zisa Palace, Palermo, Italy. 3D representation of the inversion model, from a front point of view (left picture) and rear (right picture). Plane **A**—external wall surface. Plane **B**—investigation depth. Plane **C**—internal wall mosaic surface. Green and blue colors indicate the areas of low resistivity corresponding to the detected presence of moisture. After Fiandaca et al. [70] (reprinted with copyright permission).

An innovative and challenging in-situ investigation of wall mosaics was carried out using electrical resistivity tomography (ERT) [70]. The method was applied to a precious wall mosaic in the fountain room of the Zisa Palace, Palermo (Italy), in 2009. A 3D map of electrical resistivity distribution inside the wall was realized. The lower resistivity zone was interpreted as the moisture infiltration in the middle of the wall (Figure 5). It corresponds to the location of the water-pipe of the fountain. The method proved its effectiveness in the detection of the problem and showed clear readability and interpretation of the results. This method is still scarcely applied in the field of cultural heritage, due to the invasivity of mini electrodes. As shown by the first experimental results, if the problem of invasivity can be solved, this method shows strong potential for subsurface and deep wall structure evaluation.

### 3.5. Laboratory Simulation Studies

Electronic speckle pattern interferometry (ESPI) was applied to a custom-built mosaic model with known defects [71]. The decoration of the mosaic model was realized using glass paste tesserae; therefore, it was representative of a wall mosaic. The ESPI system was capable of revealing the two known defects (detachments at different depth levels), showing limitations due to partial transparency of glass tesserae, characteristic for wall mosaics, and high sensitivity of the system to external vibrations. In scientific literature, it is the first documented application of ESPI to a wall mosaic in 1998. The reader is invited to consult the relative sources for a more comprehensive treatment of the subject [39,40,41,42,71].

More laboratory studies are dedicated to the combination of holographic interferometry and infrared thermography in wall mosaic diagnostics. Laboratory tests were performed on plastered mosaics and revealing hidden layers and differences by means of holographic interferometry and transient thermography in 2012 [72]. The search of the most appropriate methods in the detection of plastered mosaics is important for the cases like Hagia Sophia mosaics, the reader can refer to the literature sources cited above [61,62,63,64,65]. Laboratory experimentation confirmed the efficiency and complementarity of the two techniques. The transient thermography provided results concerning the identification of the different subsurface tesserae areas of the plastered panel, differentiating between stone, metal leaf, and glass paste. Holographic interferometry identified the precise demarcation lines of these areas, which were not possible to perceive for all the hidden areas by the thermographic survey.

**Figure 6:**
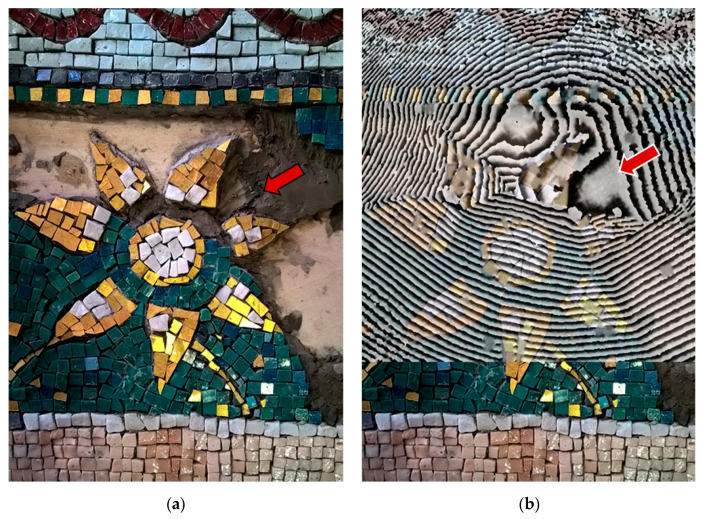
Digital holographic speckle pattern interferometry (DHSPI) results in a custom-built mosaic model. Efficient recognition of mortar inhomogeneity, stitches, and cracks: (**a**) sample preparation in progress, detail of mortar application with a defect; (**b**) Interferogram at 170 s after Δt = 3.1 °C thermal excitation on a completed mosaic model, overlap with the photographic documentation of the sample preparation (25% transparency mode). After Chaban et al. [73].

Complementary approach by DHSPI-SIRT and HSR was studied in 2018 on a custom-built mosaic model with known defects and subsurface mortar inhomogeneities, covered by glass paste, golden leaf, and stone tesserae [73]. The DHSPI system was capable of detecting the stitches between mortar portions with slightly variable composition, as well as the presence of voids, detachments, and cracks underneath the mosaic decoration layer (Figure 6). The differences in the mortar (application mode, slight changes in the proportions of ingredients) were observed on an interferogram starting from very low thermal excitations (less than 1 °C). All the applied excitation values were below Δt = 3.5 °C, within the limits of allowed indoor museum temperature fluctuations. The SIRT results acquired simultaneously to DHSPI, did not reveal internal mortar characteristics but were useful to locate and to confirm the presence of detachments and, through subsequent data analysis, the presence of hidden metallic elements (nails, a mounting plate) and wooden sticks. Different studies on advanced potentials of active infrared thermography for revealing hidden layers and deterioration problems, e.g., through different acquisition and data processing modes [67] or the automatic detection of inhomogeneities [74], are available too.

**Figure 7:**
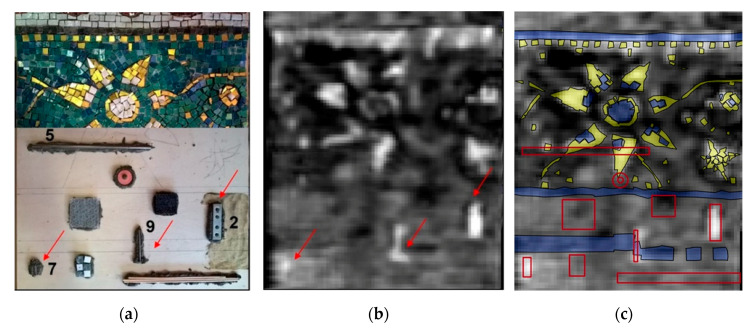
Holographic subsurface radar (HSR) results on a custom-built mosaic model: (**a**) Visible image showing the location of defects in the subsurface structure; (**b**) Rascan image at 6.7–6.8 GHz, where the red arrows indicate the location of the detected embedded metallic elements; (**c**) Rascan image with the location of defects (contour in red), stone tesserae (blue) and golden leaf tesserae (yellow). After Chaban et al. [73].

Laboratory tests by DHSPI-SIRT were complemented by the HSR method [73]. This technique was primarily applied on wall mosaic panels in the church of Santa Maria dell’Ammiraglio (Palermo, Italy) [75], though it did not provide useful results. The signal was reflected from the irregular mosaic surface, made of gold leaf tesserae, without the possibility to provide information about the underlying layers. The absence of informative in-situ results led to a series of laboratory experiments. The subsequent tests confirmed the absence of HSR signal penetration underneath the gold leaf background but proved its capability to detect metallic elements underneath glass paste and stone tesserae, even at irregular surfaces [73]. The HSR laboratory results are shown in Figure 7.

**Figure 8:**
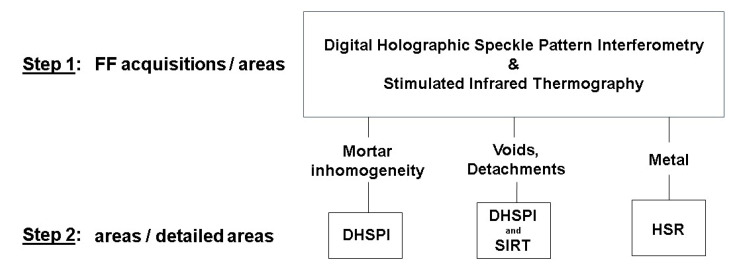
A proposed complementary DHSPI-SIRT and HSR approach for a complex subsurface structural evaluation of wall mosaics, based on the first laboratory experimental results. FF—full field; DHSPI—Digital Holographic Speckle Pattern Interferometry; SIRT—Stimulated Infrared Thermography; HSR—Holographic Subsurface Radar. After Chaban et al. [73].

A complementary approach by DHSPI-SIRT and HSR allowed for the detection of mortar subsurface structure (by DHSPI), detachments (by DHSPI-SRT), and buried metallic elements (by HSR) [73]. Each of these methods showed certain advantages and limitations, and the proposed combination can be a starting point and a reference study for future frontiers in the non-invasive techniques development study. A possible workflow for non-invasive mosaic subsurface structural evaluation, based on laboratory results, was proposed using DHSPI-SIRT and HSR (see Figure 8).

**Figure 9:**
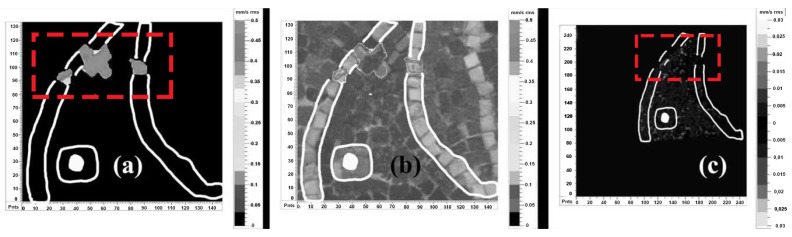
Scanning laser doppler vibrometry (SLDV) results in a custom-built mosaic model. Vibration maps of sample mosaic at 1525 Hz (**a**) a grayscale image representing the vibrational amplitude before restoration, the areas of detached tesserae show higher amplitude, corresponding to lighter shades of grey; (**b**) contour view before restoration; (**c**) amplitude after restoration, showing the more homogeneous distribution of amplitude values along the scanned surface. After Castellini et al. [76] (reprinted with copyright permission).

Laboratory tests in 2003 include applications of another promising technique scanning laser doppler vibrometry (SLDV) to series of art objects, including a mock-up of a newly made mosaic with an area of detached glass paste tesserae [76]. With acoustic excitation, before and after gluing, the detached tesserae on the mosaic model showed the promising results of the technique (Figure 9). This revealed the difference in vibration amplitude before and after gluing the tesserae. The examined mosaic model was held in front of the SLDV, and further studies are to be carried out to verify this condition at different angles.

## 4. Conclusions and Future Directions

The review of scientific publications dedicated to the complex non-invasive study of wall mosaics showed that a variety of methods is available, which are already or potentially applicable in-situ, at their current development stage or, hopefully, through further improvements, in the next future. The applicability of main on-site non-invasive techniques to the complex evaluation of wall mosaics is illustrated in Table 1. Based on the results published by the international scientific community, the available non-invasive techniques are assessed in terms of their applicability and informativity for a mosaic wall decoration. Table 2 summarizes the advantages and limitations of each considered technique.

The most discussed research questions in wall mosaics study are conservation problems like moisture rise, cracks, detachments, assessment of conservation treatments, and historical issues, e.g., the presence of hidden valuable mosaic layers. The most informative in-situ acquisitions, described in scientific literature, include the well-known IRT and GPR. Laboratory studies show the growth of interest in the development of new portable non-contact full-field methods, where a strong potentiality is shown by DHSPI, DHSPI-SIRT, SLDV, as well as by HSR (with certain limitations) etc.. The literature review has shown a strong interest in structural deterioration and a specific issue of plastered (intentionally covered) wall mosaics. On the other hand, studies dedicated to reshuffled/restored mosaics, with the identification of presumably original and transferred/restored areas, in authors’ awareness, are not present in the scientific literature. Further studies have to be carried out also on gold leaf backgrounds, which are very common in Byzantine wall mosaics and where the application of surface penetrating radar and infrared thermography techniques is limited (the radiation is reflected from the metallic surface without the possibility to obtain information about the underlying layers).

**Figure 10:**
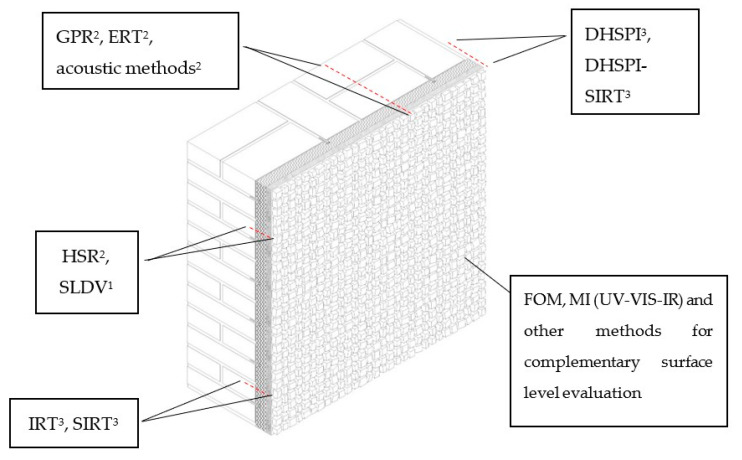
Complementarity of different techniques to a complex (depth-resolved) non-invasive study of wall mosaics, where: GPR—Ground Penetrating Radar, DHSPI—Digital Holographic Speckle Pattern Interferometry, FOM—Fiber Optics Microscopy, MI—Multispectral Imaging, IRT—Infrared Thermography, SIRT—Stimulated Infrared Thermography, SLDV—Scanning Laser Doppler Vibrometry; HSR—Holographic Subsurface Radar. ^1^ non-contact spot analysis; ^2^ method that requires physical contact to the surface, spot/line acquisition; ^3^ non-contact full-field.

Through the overview and comparison of documented in-situ applications, it can be confirmed that research questions and the conservation problems differ from mosaic to a mosaic. A unique, universally applicable method to wall mosaic decoration, providing information at all the depth levels, does not exist. Every method is characterized by certain advantages and limitations by a possible penetration depth, resolution parameters, and acquisition requirements. Therefore, the only universal rule is that a complementary use of at least two or more informative scientific methods is essential (Figure 10).

The most critical parameters in choosing a method for in-situ wall mosaic diagnostics are their non-destructivity, portability, efficiency in answering the research question, and (possibly) non-contact operation. If these requirements are satisfied, the method, characterized by a fast, easy, and full-field acquisition, should be preferred. Further aspects to be considered include time management, access to the site and acquisition conditions, team expertise, available instrumentation, and costs.

Further frontiers of the development of these and new methods are represented by increasing the informativity, feasibility, and timing, while decreasing invasivity, contact operation, and costs. There is a strong demand for further development of full-field visualization and non-contact methods, providing a comprehensive structural evaluation approach at all the depth levels. This non-invasive and non-destructive structural evaluation, in an ideal case, will be performed in automated monitoring mode. This will support the achievement of the fundamental goal in the field of cultural heritage diagnostics: timely and efficient detection, prevention of deterioration, limiting loss, microsampling, or any other impact and keeping alterations to a minimum, as an exception, or finally to zero.

## Figures and Tables

**Table 1:** Applicability of non-invasive techniques on wall mosaics.

Methods	Characteristics			Applicability		Resolution		Case Studies, Lab Tests on Wall Mosaics * (References)
	Portable	Non-Contact	Full-Field	Irregular, Inhomogeneous Surfaces	Gold Leaf Tesserae	Shallow Depth	Deep Structure	
GPR	+	−	−	+/−	−	+/−	+	[61,62,63,64,65,69]
IRT/SIRT	+	+	+	+	−	+	+/−	[61,62,64,65,66,67,72,73,74,75]
DHSPI/DHSPI-SIRT	+	+	+	(+)	(+)	(+)	o	[73,75]
HSR	+	−	−	(−)	(−)	(+)	o	[73,75]
ERT	+	−	−	+	+	+	+/−	[70]
acoustic	+	−	−	+	o	+	+	[69]
SLDV	+	+	−	(+)	o	(+)	o	[76]
other (MI/FOM)	+	+	+/−	+	+	−	−	[61,62,75]

“()”: only laboratory simulation results available; “+”: applicable and informative; “−“: not applicable and/or not informative; “+/**−**“: applicable with limitations; “o”: no documented case studies reported in literature. * only references dealing with applications on wall mosaics are reported in this table.

**Table 2:** Advantages and limitations of portable NDT for precious wall mosaics.

Methods	Achievements and Advantages	Limitations and Issues to Solve
GPR	Optimal technique for revealing deep wall structural defects, possibility of quantitative analysis (depth target estimation)	Contact operation, line acquisition, low resolution in shallow subsurface, signal attenuation (by moisture, salts, highly conductive materials etc.)
IRT/SIRT	Non-contact and full-field acquisition, instant visualization, easy interpretation, can be coupled to other techniques for complementary information	Low informativity if thermal gradient is not present, influence of thermal emissivity values on the mosaic surface
DHSPI/DHSPI-SIRT	Non-contact full-field acquisition, instant visualization, high resolution, detection of defects within the subsurface (voids, cracks), inhomogeneities and differences in mortar structure, can be coupled to other techniques for complementary information	High sensitivity to surrounding vibrations, sensitivity only in the direction of the optical axis of the system; bibliographic sources on in-situ tests are still missing
HSR	High efficiency in the detection of metallic elements and humidity; possibility to estimate the depth, reconstruct the exact horizontal dimensions and shape of the detected targets	Contact operation, line acquisition, not applicable on gold leaf tesserae; results high influenced by the accuracy in the acquisition; further laboratory and in-situ tests are still missing
ERT	Subsurface and deep wall structural evaluation, possibility of 2D/3D representation of resistivity values, efficiency in detectability of moisture areas	Not applicable on precious wall mosaics due to invasivity, bibliographic sources on further laboratory and in-situ tests are still missing
Acoustic (sonic/ultrasonic)	Subsurface and deep wall structural evaluation, possibility of 2D/3D representation of sonic/ultrasonic velocity values, efficiency in assessment of grouting	Contact operation, spot acquisition, not applicable on deteriorated surfaces, access to both sides of the wall for in-depth visualization
SLDV	Non-contact operation, efficient in detectability of shallow subsurface detachments	Spot acquisition, bibliographic sources on further laboratory and in-situ tests are still missing
other (MI/FOM)	Non-contact and full-field, high resolution	Low penetration (only surface analysis)
